# Using Machine Learning Technologies in Pressure Injury Management: Systematic Review

**DOI:** 10.2196/25704

**Published:** 2021-03-10

**Authors:** Mengyao Jiang, Yuxia Ma, Siyi Guo, Liuqi Jin, Lin Lv, Lin Han, Ning An

**Affiliations:** 1 Evidence-based Nursing Center School of Nursing Lanzhou University Lanzhou China; 2 Key Laboratory of Knowledge Engineering with Big Data of the Ministry of Education School of Computer Science and Information Engineering Hefei University of Technology Hefei China; 3 Wound and Ostomy Center Outpatient Department Gansu Provincial Hospital Lanzhou China; 4 Department of Nursing Gansu Provincial Hospital Lanzhou China

**Keywords:** pressure injuries, pressure ulcer, pressure sore, pressure damage, decubitus ulcer, decubitus sore, bedsore, artificial intelligence, machine learning, neural network, support vector machine, natural language processing, Naive Bayes, bayesian learning, support vector, random forest, boosting, deep learning, machine intelligence, computational intelligence, computer reasoning, management, systematic review

## Abstract

**Background:**

Pressure injury (PI) is a common and preventable problem, yet it is a challenge for at least two reasons. First, the nurse shortage is a worldwide phenomenon. Second, the majority of nurses have insufficient PI-related knowledge. Machine learning (ML) technologies can contribute to lessening the burden on medical staff by improving the prognosis and diagnostic accuracy of PI. To the best of our knowledge, there is no existing systematic review that evaluates how the current ML technologies are being used in PI management.

**Objective:**

The objective of this review was to synthesize and evaluate the literature regarding the use of ML technologies in PI management, and identify their strengths and weaknesses, as well as to identify improvement opportunities for future research and practice.

**Methods:**

We conducted an extensive search on PubMed, EMBASE, Web of Science, Cumulative Index to Nursing and Allied Health Literature (CINAHL), Cochrane Library, China National Knowledge Infrastructure (CNKI), the Wanfang database, the VIP database, and the China Biomedical Literature Database (CBM) to identify relevant articles. Searches were performed in June 2020. Two independent investigators conducted study selection, data extraction, and quality appraisal. Risk of bias was assessed using the Prediction model Risk Of Bias ASsessment Tool (PROBAST).

**Results:**

A total of 32 articles met the inclusion criteria. Twelve of those articles (38%) reported using ML technologies to develop predictive models to identify risk factors, 11 (34%) reported using them in posture detection and recognition, and 9 (28%) reported using them in image analysis for tissue classification and measurement of PI wounds. These articles presented various algorithms and measured outcomes. The overall risk of bias was judged as high.

**Conclusions:**

There is an array of emerging ML technologies being used in PI management, and their results in the laboratory show great promise. Future research should apply these technologies on a large scale with clinical data to further verify and improve their effectiveness, as well as to improve the methodological quality.

## Introduction

Pressure injury (PI) is a significant indicator of the quality of care and a substantial burden on the public health system and the economy [1,2]. PI is a common but potentially preventable problem; however, current PI management is far from satisfactory. PI incidence and prevalence in the intensive care unit (ICU) were reported to be 10.0% to 25.9% and 16.9% to 23.8%, respectively [3]. The prevalence of PI in acute care settings ranged from 6% to 18.5% [4] and the hospital-acquired PI prevalence was 8.5% [5]. As for long-term care facilities, the PI prevalence was 27% in Italy [6] and 9.6% in Japan [7]. The overall prevalence of PI in the United States decreased from 13.5% in 2006 to 9.3% in 2015 [8]. Also, 95% of PIs are avoidable [9]. Nurses are primarily responsible for preventing PIs [10]. Several surveys have revealed that the majority of nurses, internationally, have insufficient knowledge of PI [11-14]. Besides, the global nursing shortage is a well-known fact [15]. Also, the most universally used PI risk assessment tool—the Braden scale—is subjective and inaccurate [16]. In a nutshell, medical practitioners need better PI management tools.

Artificial intelligence (AI) has been exerting a positive impact on daily living [17]. Moreover, machine learning (ML) is a way to achieve AI. Over the past two decades, ML has progressed from a laboratory curiosity to practical tools commonly applied in the medical field [18,19]. ML will continue to contribute to improving prognosis and diagnostic accuracies, even potentially taking on some of the work of medical practitioners’ [20,21]. While researchers have developed various novel methods for PI management [22], there is no systematic review to our knowledge that evaluates current ML technologies used in PI management.

The objective of this paper was to synthesize and evaluate the nascent literature on the use of ML technologies in PI management, noting the strengths and weaknesses of the studies, and identify improvement opportunities for future research and practice.

## Methods

### Protocol

This review is reported according to the PRISMA (Preferred Reporting Items for Systematic Reviews and Meta-Analyses) statement [23].

### Search Strategy

We conducted a systematic search of nine health science databases: PubMed, EMBASE, Web of Science, Cumulative Index to Nursing and Allied Health Literature (CINAHL), Cochrane Library, China National Knowledge Infrastructure (CNKI), the Wanfang database, the VIP database, and the China Biomedical Literature Database (CBM). We used Medical Subject Headings (MeSH) terms, Emtree terms, subject headings, and free text associated with the concepts of ML and PI. Searches were performed in June 2020. We also undertook a manual search of the reference list of all potentially eligible studies. Textbox 1 shows the search strategy that was used.

**Search strategy and search terms used.**

- #1 pressure ulcer* OR pressure injur* OR pressure sore* OR pressure damage OR decubitus ulcer* OR decubitus sore* OR bedsore* OR bed sore*

AND

- #2 artificial intelligence OR machine learning OR neural network* OR support vector machine OR natural language processing OR Naive Bayes OR bayesian learning OR support vector* OR random forest* OR boosting OR deep learning OR machine intelligence OR computational intelligence OR computer reasoning

### Inclusion and Exclusion Criteria

This review included studies that met the following criteria: (1) used a method related to ML technologies (including support vector machine, k-nearest neighbor [KNN], decision tree [DT], convolutional neural network, Bayesian network model, and logistic regression) in PI management, and (2) was published in English or Chinese. We excluded studies that met any of the following criteria: (1) review papers, opinion papers, editorials, discussion papers, dissertations, or conference abstracts; (2) papers on PI education; (3) papers about PI in animals; (4) papers lacking an outcome; and (5) papers without explicit algorithms.

### Study Selection Methods

Two independent investigators screened titles and abstracts using the eligibility criteria. They then obtained full-text versions of all potential articles and scrutinized the full texts independently. Any discrepancies about study inclusion were resolved through discussion or by referral to a third investigator.

### Data Extraction

Data were extracted from all identified studies using a predefined format. Variables included the first author, year of publication, country, aim, subject, algorithm used, study outcomes, performance of the algorithm, and findings. One investigator extracted the information into a standard data extraction sheet and a second investigator cross-checked the entries. Any disagreements were resolved via discussion.

### Quality Appraisal

The methodological quality of the included studies was assessed independently by two investigators using the Prediction model Risk Of Bias ASsessment Tool (PROBAST) [24]. Disagreements were resolved by discussion. The PROBAST was designed to assess the risk of bias and applicability of diagnostic and prognostic prediction model studies, and it includes 20 signaling questions to judge the risk of bias from four domains (participants, predictors, outcome, and analysis). The risk of bias is judged as low, high, or unclear. If one domain is found to have a high risk of bias, the overall risk of bias is judged as high. Similarly, if one domain is assessed as unclear, the overall risk of bias is judged as unclear even if all other domains are assessed to have a low risk of bias.

## Results

### Study Process

**Figure 1:**
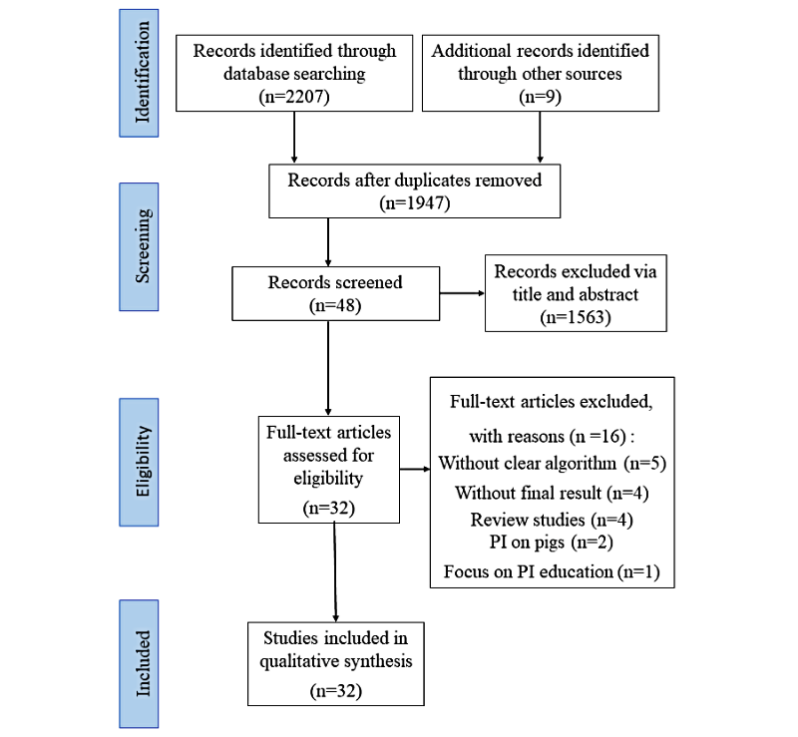
PRISMA (Preferred Reporting Items for Systematic Reviews and Meta-Analyses) flow diagram of the inclusion process. PI: pressure injury.

Our initial search retrieved 2207 published articles, of which 269 were duplicates. After screening titles and abstracts, the full texts of 48 articles were obtained and assessed for potential eligibility. Of those 48 articles, 16 did not fulfill the inclusion criteria. The reasons for studies being ineligible were as follows: (1) lacking a clear algorithm (n=5); (2) lacking a result (n=4); (3) review studies (n=4); (4) studies in pigs (n=2); and (5) study on PI education (n=1). Finally, a total of 32 studies were eligible for our research (see Figure 1).

### Characteristics of Included Studies

The articles that were included in our analysis were published between 2007 and 2020 and were undertaken in the United States [25-35], China [36-44], Spain [45-50], Japan [51,52], Italy [53,54], Korea [55], and Greece [56]. According to the applied area of the included studies, we divided the articles into three components: predictive model (12 studies), posture recognition (11 studies), and image analysis (9 studies). The characteristics of the included studies are presented in Multimedia Appendix 1.

**Figure 2:**
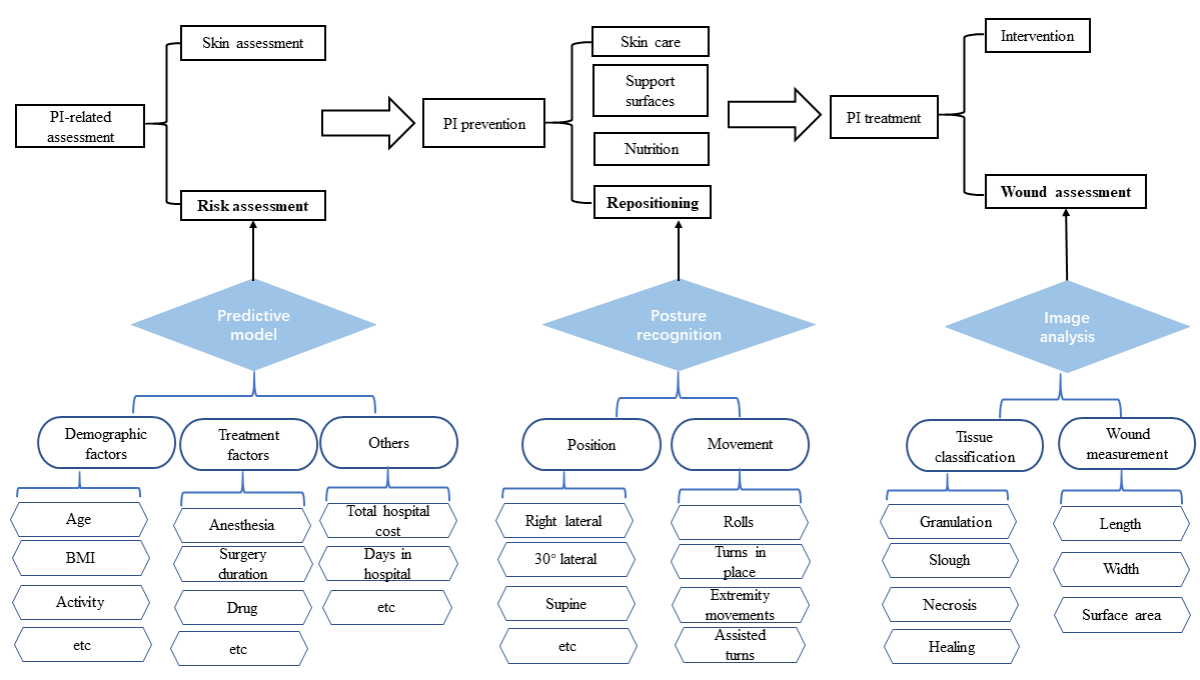
The roles of machine learning technologies used in pressure injury (PI) management.

Figure 2 shows the roles of the three components in the PI management process:

- Predictive model: when a patient is admitted into the hospital, a nurse needs to perform PI-related assessments—skin assessment and risk assessment. The predictive model is used to identify related risk factors.
- Posture recognition: when a patient is determined to be at risk, according to PI guidelines, proper measures such as repositioning, nutrition, support surfaces, and skin care need to be taken to prevent PI. The posture recognition can be used in the repositioning to help nurses to detect and classify the patient’s position and movement.
- Image analysis: when a PI occurs, it is necessary to do wound assessment prior to treating the wounds. The image analysis can help to classify the wound tissue and measure the wound size.

The performance indicators of ML algorithms include sensitivity, specificity, precision, accuracy, F score, positive predictive value, negative predictive value, geometric mean, false-positive rate, run time, and so on. Multimedia Appendix 2 shows the detailed results of the included studies.

### Predictive Model

Twelve studies explored PI risk factors by data mining from the electronic health records (EHRs) of patients. The patients included in the studies were from a variety of settings: ICU (3 studies); operating room (2 studies); long-term care facilities (1 study); acute care hospital (1 study); orthopedic department (1 study); oncology department (1 study); end-of-life care (1 study); medical-surgical, critical care, and step-down units (1 study); and with mobility-related disabilities (1 study). The number of EHRs ranged from 147 to 125,213. The identified risk factors were different due to diverse input variables. In the majority of included studies, the PI percentage (the number of patients with PI/the number of total patients) of the data sets analyzed was imbalanced, and the minimum was 0.6% (51/8286). The accuracy ranged from 63.0% to 90.0%, the sensitivity ranged from 47.8% to 84.8%, and the specificity ranged from 70.3% to 94.7%. The DT algorithm was a typical data mining approach.

### Posture Recognition

Eleven studies were concerned with posture identification by analyzing the pressure distribution of the body to achieve a robust assessment. Regarding the subjects of posture recognition, one study focused on wheelchair users [38], while the others looked at bed bound patients. The number of sensors was between 4 and 8192, and the number of subjects ranged from 2 to 58. Of the 11 studies, 10 studies detected and classified different postures or movements of a person and one study classified the bed inclination [31]. The common postures detected were supine, right lateral, and left lateral.

All articles reported on accuracy, which ranged from 49.1% to 100%. The difference in run times among different algorithms was quite large, from 0.04 seconds to 320.34 seconds. No articles reported on specificity. The sensitivity ranged from 62.0% to 100%, and the precision ranged from 65.0% to 100%. All eight studies applied the KNN algorithm in the processing of pressure sensor data.

### Image Analysis

Nine studies conducted PI wounds’ tissue segmentation and measurement using ML algorithms. We included studies that only analyzed PI images and excluded those involving the wound images of diabetes foot ulcers or venous leg ulcers. The number of digital images ranged from 14 to 193. Three articles were written by Veredas et al [46,48,49] using the same 113 color images to achieve tissue classification. Because different algorithms were used, we considered these three articles as independent research. Furthermore, the number of tissue segmentations ranged from 3 to 6. The most common PI wound tissue classifications were granulation, slough, and necrosis. One study developed an image processing algorithm that automatically measured the PI size [30]. The accuracy ranged from 78.3% to 92.0%, the sensitivity ranged from 61.7% to 99.9%, and the specificity ranged from 93.9% to 99.8%. Convolutional neural network algorithms, as deep learning architectures, were often used in medical image analysis in recent years.

### Risk of Bias

**Figure 3:**
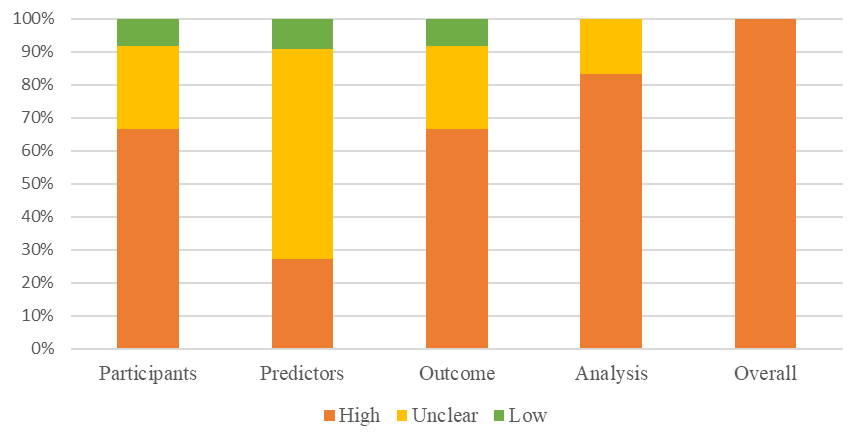
Risk of bias assessment for the predictive model studies.

The PROBAST was used to assess the risk of bias of the predictive model studies from four domains (participants, predictors, outcome, and analysis). However, the PROBAST was not suitable for the posture recognition and image analysis studies; to the best of our knowledge, there is still no appropriate tool to assess these engineering articles. The overall risk of bias of all of the predictive model studies was judged as high, and there was no low risk in the analysis domain (Figure 3).

## Discussion

### Principal Findings

Our systematic review provided a broad overview of the ML technologies applied to PI management. After study selection, we were able to categorize these technologies into three components: predictive model, posture recognition, and image analysis. We discuss these different components in detail below.

#### Component 1: Predictive Model

The predictive model studies were all retrospective studies that analyzed the EHRs of patients to develop a prediction model via data mining techniques. The objective of the predictive model was to (1) identify the PI risk factors so that nurses could take customized preventive measures to arrest the PI progression, or (2) compare different algorithm performances and interpretability in constructing a predictive model. Even though the data sets were often imbalanced, Setoguchi et al [51] suggested that an alternating DT algorithm could effectively analyze highly imbalanced data. Shi et al [57] identified 22 empirically derived predictive models for PI risk using traditional statistical techniques. Compared with the previous predictive models, these advanced models can use the information available in EHRs rather than require investigators to input information into a questionnaire, and they can handle a large volume of various data at a faster velocity. Relative to the 2019 international guideline [1], we found a gap between the ML models and the empirical models. The risk factors mentioned in the guideline are mainly patient characteristics (eg, older age, spinal cord injuries, diabetes, incontinence, impaired sensory perception, etc) and treatment plan (eg, duration of surgery, anesthesia, use of vasopressors, etc). By employing ML models using data from patients’ EHRs, Moon and Lee [55] found that the total hospital cost was associated with PIs, which had not been revealed by the guideline. However, it must be noted that these ML-based predictive models were lacking external validation. The results we got from one database had not been validated in temporal or spatial difference. Clearly, providing external validation for these models should be a focus of future research.

#### Component 2: Posture Recognition

PIs (also called bedsores) are common among bedridden older patients. However, the subjects in the included research studies were all healthy adults of different weights rather than patients at high risk for PIs. The research to test the ML technologies’ performance was all conducted in the laboratory. In other words, these technologies are still in the development phase and have not transitioned from bench to bedside. The current research focused simply on posture detection, and the majority of repositioning recommendations from the 2019 international guideline were based on expert opinion. Future research should combine posture recognition with the predictive model to develop the most effective repositioning schedules. For example, it is generally acknowledged that patients should be repositioned or mobilized every 2 hours. For a high-risk patient, it may be better to reposition every hour, while a low-risk patient may need to be repositioned every 3 hours. When it is time to change the patient’s position, the related alarm will alert the nurse to help the patient to reposition, thus lightening the clinical nurse’s workload.

#### Component 3: Image Analysis

It is worth mentioning that 6 of 9 (67%) studies were conducted in Spain. All three articles of Veredas et al (45,47,48) analyzed 113 digital images of PI of patients with home-care assistance, and we can assume that these were the same subjects; however, it is quite interesting to note that the images in the article published in 2010 were taken with a Canon digital camera, while the images in the 2015 article were taken with a Sony digital camera. In the real world, PI wounds are always irregular in shape, and it is inaccurate and unreliable to measure the size of the PI wound by multiplying length and width [58]. The computer-aided measurement system can offer an objective and efficient result. Using a photo of the PI wound, it is convenient and possible to analyze the characteristics of the lesion by the size and color of the ulcer, which helps clinicians monitor the developing and healing process of PI. Note that these subjects of image analysis are visible wounds, which are always stage IV—the severest PIs. Certainly, we do not want to see the most terrible situation happen, and thus future research is needed to optimize technologies so that we can assess PIs in their early stage via microclimate (eg, moisture, temperature, etc), not just via images. The current research is focused on classifying the wound tissue, and it is necessary to combine the percentage of the different tissue with the grading of PI to define the severity of PI. It is better to rely on objective indicators than to rely on human experience.

### Future Research

PI management should be a holistic process, but the current research in these three components is separate. We’ll use the case of a patient admitted to hospital to illustrate. First, according to the predictive model, we rated the patient as low risk. The repositioning schedule was implemented as the low risk required. Unfortunately, the patient developed PI, so we needed to assess the PI wound. The ML technologies on the predictive model and posture recognition need feedback from the PI wound image analysis to improve their performance. However, the research in these three components was conducted in different populations in different locations at different times. This point should be explored in future research.

The results on the risk of bias, surprisingly, were far from satisfactory. Similar to the research of Nagendran et al [59], the analysis domain was the major deficiency. More attention needs to be paid to the methodological quality of predictive model studies. The participants in posture recognition studies were healthy volunteers and the subjects in image analysis studies were images, so we could not judge these types of articles as medical research. There is a growing literature on interdisciplinary research such as in the fields of engineering and medicine. It is essential to develop a tool to assess the methodological quality of the relevant articles.

In summary, ML technologies furnish new alternatives to PI management. Given the global shortage of professional nurses and PI-related knowledge deficit, ML technologies will significantly reduce the burden on frontline clinicians and help to improve the quality of care, as Obermeyer and Emanuel [20] pointed out in 2016. However, because the current technologies only cover three components of PI management, there is a marked lack of novel technologies to assess potentially healthy skin, to achieve better skin care, to manage nutrition status, and to create intelligent support surfaces. Besides, IBM has discovered that its powerful technology is no match for the messy reality of today’s health care system [60]. There is still a long way to go to integrate ML technologies into clinical care practices.

It is important to acknowledge some limitations. First, we only include articles published in English and Chinese. It will be better to include other language research for representing the current evidence. Second, due to the various aims and outcomes of the included studies, the quantitative synthesis has not been performed to obtain a direct result. Third, the aim of our review was to survey the current status of ML algorithms applied in PI management, so the eligibility criteria were defined broadly. After study selection, we found the related research can be divided into three components. We have no specific criteria for one component. Hence, under the guidance of our findings, future research can define detailed eligibility criteria.

### Conclusions

The study results from various laboratory settings show an array of ML technologies with potential uses in PI management. Future research should apply these technologies on a large scale with clinical data to verify their effectiveness, enhance their performance, and improve methodological quality.

## Appendix

Multimedia Appendix 1 The characteristics of the included studies.

Multimedia Appendix 2 The detailed performance measurements of machine learning technologies in the included studies.

## Abbreviations

AI: artificial intelligence
CBM: China Biomedical Literature Database
CINAHL: Cumulative Index to Nursing and Allied Health Literature
CNKI: China National Knowledge Infrastructure
DT: decision tree
EHR: electronic health record
ICU: intensive care unit
KNN: k-nearest neighbor
MeSH: Medical Subject Headings
ML: machine learning
PI: pressure injury
PRISMA: Preferred Reporting Items for Systematic Reviews and Meta-Analyses
PROBAST: Prediction model Risk Of Bias ASsessment Tool
