# Supplementary material for: Using Machine Learning Technologies in Pressure Injury Management: Systematic Review
Source: JMIR Med Inform. 2021 Mar 10;9(3):e25704. doi: 10.2196/25704 (PMC7991995; doi:10.2196/25704)
Supplement: Multimedia Appendix 1 [file medinform_v9i3e25704_app1.docx]

**Multimedia Appendix 1.** The characteristics of the included studies.

Table 5 detail results of predictive model studies

| No. | author | year | country | algorithm | training | testing | accuracy | sensitivity | specificity | F-score | g-means | PPV | NPV |
| --- | --- | --- | --- | --- | --- | --- | --- | --- | --- | --- | --- | --- | --- |
| 1 | Su, Chao-Ton | 2012 | China | MTS | 75% | 25% | NR | 0.763 | 0.886 | 0.377 | 0.822 | NR | NR |
|  |  |  |  | SVM |  |  | NR | 0.667 | 0.947 | 0.610 | 0.806 | NR | NR |
|  |  |  |  | DT |  |  | NR | 0.500 | 0.981 | 0.532 | 0.700 | NR | NR |
|  |  |  |  | LR |  |  | NR | 0.625 | 0.988 | 0.670 | 0.786 | NR | NR |
| 2 | Dai, L | 2012 | China | NN | 70% | 15% | 90% | NR | NR | NR | NR | NR | NR |
| 3 | Raju, D. | 2015 | USA | RF | 90% | 10% | 0.830 | NR | NR | NR | NR | NR | NR |
|  |  |  |  | LR |  |  | 0.820 | NR | NR | NR | NR | NR | NR |
|  |  |  |  | MARS |  |  | 0.780 | NR | NR | NR | NR | NR | NR |
|  |  |  |  | DT |  |  | 0.630 | NR | NR | NR | NR | NR | NR |
| 4 | Setoguchi, Y. | 2016 | Japan | DT | NR | NR | 0.721 | 0.793 | 0.721 | NR | NR | NR | NR |
| 5 | Deng, X.H | 2016 | China | DT | NR | NR | NR | 0.809 | 0.703 | NR | NR | NR | NR |
| 6 | Kaewprag, P. | 2017 | USA | Bayesian networks | 67% | 33% | 0.819 | 0.478 | 0.895 | NR | 0.618 | 0.274 | 0.954 |
| 7 | Chen, H. L. | 2017 | China | ANN | 70% | 30% | 0.815 | NR | NR | NR | NR | NR | NR |
| 8 | Moon, M | 2017 | Korea | DT | 90% | 10% | 0.804 | 0.820 | 0.787 | NR | 0.803 | NR | NR |
| 9 | Alderden, J. | 2018 | USA | RT | 67% | 33% | 0.790 | NR | NR | NR | NR | NR | NR |
| 10 | Yang, Q | 2019 | China | DT | NR | NR | NR | 0.848 | 0.747 | NR | NR | NR | NR |
| 11 | Li, H. L. | 2019 | China | DT | NR | NR | 0.772 | 0.796 | 0.748 | NR | 0.770 | NR | NR |
|  |  |  |  | NN |  |  | 0.781 | 0.814 | 0.749 | NR | 0.779 | NR | NR |
|  |  |  |  | SVM |  |  | 0.793 | 0.810 | 0.788 | NR | 0.798 | NR | NR |
| 12 | Sprigle, S | 2020 | USA | Gradient boosting | NR | NR | NR | 0.700 | 0.920 | NR | NR | NR | NR |

Table 6 detail results of posture recognition studies

| No. | author | year | country | algorithm | training | testing | accuracy | sensitivity | specificity | precision | F-score | run-time(s) |
| --- | --- | --- | --- | --- | --- | --- | --- | --- | --- | --- | --- | --- |
| 1 | Barsocchi, P. | 2012 | Italy | SVM | NR | NR | 90.90% | NR | NR | NR | NR | NR |
|  |  |  |  | k-NN |  |  | 100% | NR | NR | NR | NR | NR |
| 2 | Baran Pouyan, M. | 2014 | USA | k-NN | NR | NR | 80.3% | 0.80 | NR | 0.81 | 0.80 | NR |
|  |  |  |  | Naïve-bayes |  |  | 80.3% | 0.62 | NR | 0.65 | 0.63 | NR |
|  |  |  |  | C4.5 |  |  | 80.3% | 0.76 | NR | 0.78 | 0.77 | NR |
|  |  |  |  | Part |  |  | 80.3% | 0.74 | NR | 0.75 | 0.74 | NR |
| 3 | Xu, X. | 2016 | China | k-NN-planar | NR | NR | 90.26% | 0.902 | NR | NR | NR | 8.07 |
|  |  |  |  | k-NN-polar |  |  | 89.26% | 0.892 | NR | NR | NR | 15.96 |
|  |  |  |  | k-NN-projection |  |  | 91.21% | 0.912 | NR | NR | NR | 3.27 |
| 4 | Baran Pouyan, M. | 2016 | USA | k-NN | NR | NR | 95.10% | NR | NR | NR | NR | NR |
| 5 | Heydarzadeh, M. | 2016 | USA | HoG+DNN | 70% | 30% | 98.10% | NR | NR | NR | NR | NR |
| 6 | Hsiao, R. S. | 2016 | China | Fuzzy | NR | NR | 92.09% | NR | NR | NR | NR | NR |
|  |  |  |  | SVM |  |  | 82.16% | NR | NR | NR | NR | NR |
|  |  |  |  | k-NN(k=1) |  |  | 81.10% | NR | NR | NR | NR | NR |
| 7 | Ma, C. | 2017 | China | J48 | NR | NR | 99.48% | 0.995 | NR | 0.995 | 0.995 | 1.98 |
|  |  |  |  | SVM |  |  | 79.08% | 0.736 | NR | 0.88 | 0.76 | 320.34 |
|  |  |  |  | MLP |  |  | 95.50% | 0.926 | NR | 0.926 | 0.926 | 265.46 |
|  |  |  |  | Naïve Bayes |  |  | 49.09% | 0.491 | NR | 0.585 | 0.427 | 0.24 |
|  |  |  |  | BayesNet |  |  | 94.06% | 0.941 | NR | 0.945 | 0.941 | 0.93 |
|  |  |  |  | k-NN(k=1) |  |  | 98.53% | 0.955 | NR | 0.995 | 0.995 | 0.04 |
|  |  |  |  | k-NN(k=5) |  |  | 98.52% | 0.955 | NR | 0.995 | 0.995 | 0.08 |
| 8 | Enayati, M. | 2018 | USA | k-fold | 98.30% | 1.75% | 81.00% | NR | NR | NR | NR | NR |
|  |  |  |  | LOSO |  |  | 75.00% | NR | NR | NR | NR | NR |
| 9 | Matar, G. | 2018 | Canada | ANN | 58.30% | 41.70% | 97.90% | NR | NR | NR | NR | NR |
| 10 | Duvall, J. | 2019 | USA | k-NN(k=1) | 25% | 75% | 94.90% | NR | NR | NR | NR | NR |
|  |  |  |  | k-NN(k=2) |  |  | 94.80% | NR | NR | NR | NR | NR |
|  |  |  |  | k-NN(k=3) |  |  | 95.60% | NR | NR | NR | NR | NR |
|  |  |  |  | k-NN(k=4) |  |  | 95.90% | NR | NR | NR | NR | NR |
|  |  |  |  | k-NN(k=5) |  |  | 95.90% | NR | NR | NR | NR | NR |
|  |  |  |  | k-NN(k=6) |  |  | 95.50% | NR | NR | NR | NR | NR |
|  |  |  |  | k-NN(k=7) |  |  | 95.50% | NR | NR | NR | NR | NR |
|  |  |  |  | k-NN(k=8) |  |  | 95.90% | NR | NR | NR | NR | NR |
|  |  |  |  | k-NN(k=9) |  |  | 95.60% | NR | NR | NR | NR | NR |
|  |  |  |  | k-NN(k=10) |  |  | 95.60% | NR | NR | NR | NR | NR |
|  |  |  |  | k-NN(k=11) |  |  | 95.70% | NR | NR | NR | NR | NR |
|  |  |  |  | k-NN(k=12) |  |  | 95.50% | NR | NR | NR | NR | NR |
|  |  |  |  | k-NN(k=13) |  |  | 95.80% | NR | NR | NR | NR | NR |
|  |  |  |  | k-NN(k=14) |  |  | 95.90% | NR | NR | NR | NR | NR |
|  |  |  |  | k-NN(k=15) |  |  | 95.50% | NR | NR | NR | NR | NR |
|  |  |  |  | k-NN(k=16) |  |  | 96% | NR | NR | NR | NR | NR |
|  |  |  |  | k-NN(k=17) |  |  | 95.60% | NR | NR | NR | NR | NR |
|  |  |  |  | k-NN(k=18) |  |  | 95.80% | NR | NR | NR | NR | NR |
|  |  |  |  | k-NN(k=19) |  |  | 95.40% | NR | NR | NR | NR | NR |
|  |  |  |  | k-NN(k=20) |  |  | 95.40% | NR | NR | NR | NR | NR |
|  |  |  |  | k-NN(k=21) |  |  | 95.70% | NR | NR | NR | NR | NR |
|  |  |  |  | k-NN(k=22) |  |  | 95.70% | NR | NR | NR | NR | NR |
|  |  |  |  | k-NN(k=23) |  |  | 95.30% | NR | NR | NR | NR | NR |
|  |  |  |  | k-NN(k=24) |  |  | 95.80% | NR | NR | NR | NR | NR |
|  |  |  |  | k-NN(k=25) |  |  | 95.80% | NR | NR | NR | NR | NR |
| 11 | Cicceri, G | 2020 | Italy | DNN | 85% | 15% | 99.56% | 100% | NR | 100% | 100% | NR |
|  |  |  |  | SVM |  |  | 99.32% | 98% | NR | 99% | 99% | NR |
|  |  |  |  | RF |  |  | 82.8% | 82% | NR | 70% | 75% | NR |

Table 7 detail results of image analysis studies

| No. | author | year | algorithm | training | testing | accuracy | sensitivity | specificity | precision | success | F-score | kappa | PPV | NPV | detection rate | detection prevalence | inter-rater reliabilities | Intra-rater reliability | DSC-correspondence | PAD--difference | AUC |
| --- | --- | --- | --- | --- | --- | --- | --- | --- | --- | --- | --- | --- | --- | --- | --- | --- | --- | --- | --- | --- | --- |
| 1 | Kosmopoulos, D. | 2007 | Greece | NR | NR | 78.32%-stage 1 | NR | NR | NR | NR | NR | NR | NR | NR | NR | NR | NR | NR | NR | NR | NR |
|  |  |  |  |  |  | 75.44%-stage 2 |  |  |  |  |  |  |  |  |  |  |  |  |  |  |  |
|  |  |  |  |  |  | 82.73%-stage 3 |  |  |  |  |  |  |  |  |  |  |  |  |  |  |  |
|  |  |  |  |  |  | 84.11%-stage 4 |  |  |  |  |  |  |  |  |  |  |  |  |  |  |  |
|  |  |  |  |  |  | 87.65%-white necrosis |  |  |  |  |  |  |  |  |  |  |  |  |  |  |  |
|  |  |  |  |  |  | 91.20%-black necrosis |  |  |  |  |  |  |  |  |  |  |  |  |  |  |  |
| 2 | Veredas, F. | 2010 | BCM | NR | NR | 90.3% | 75.7% | 93.9% | NR | 84.8% | NR | NR | NR | NR | NR | NR | NR | NR | NR | NR | NR |
|  |  |  | EASM |  |  | 91.1% | 77.5% | 94.4% | NR | 85.9% | NR | NR | NR | NR | NR | NR | NR | NR | NR | NR | NR |
| 3 | Veredas, F. | 2015 | k-means | 26% | 74% | 87.7% | 99.9% | 99.8% | 98.1% | NR | 73.8% | 65.8% | NR | NR | NR | NR | NR | NR | NR | NR | 94.20% |
| 4 | Noguchi, H | 2014 | Japan | NR | NR | NR | 61.7% | NR | 71.3% | NR | 59.7% | NR | NR | NR | NR | NR | NR | NR | NR | NR | NR |
| 5 | Veredas, F. | 2015 | SVM | 80% | 20% | 86.5% | 76.4% | 96.6% | NR | NR | NR | NR | 86.6% | 93.4% | 16.9% | 19.5% | NR | NR | NR | NR | NR |
|  |  |  | NN |  |  | 84.5% | 74.8% | 94.2% | NR | NR | NR | NR | 78.7% | 92.9% | 16.6% | 21.1% | NR | NR | NR | NR | NR |
|  |  |  | RF |  |  | 89.6% | 82.3% | 96.9% | NR | NR | NR | NR | 88.3% | 95.0% | 18.2% | 20.6% | NR | NR | NR | NR | NR |
| 6 | Li, D. | 2017 | SVMs | NR | NR | NR | NR | NR | NR | NR | NR | NR | NR | NR | NR | NR | 0.89--length | 0.89 | NR | NR | NR |
|  |  |  |  |  |  |  |  |  |  |  |  |  |  |  |  |  | 0.84--width |  |  |  |  |
|  |  |  |  |  |  |  |  |  |  |  |  |  |  |  |  |  | 0.86--surface area |  |  |  |  |
| 7 | Garcia-Zapirain, B. | 2018 | CNN | 60% | 40% | NR | NR | NR | NR | NR | NR | NR | NR | NR | NR | NR | NR | NR | 92% | 13% | 95% |
| 8 | Zahia, S. | 2018 | CNN | 75% | 25% | 92.01% | NR | NR | 97.31%--granulation | NR | NR | NR | NR | NR | NR | NR | NR | NR | 91.38% | NR | NR |
|  |  |  |  |  |  |  |  |  | 96.59%--necrotic |  |  |  |  |  |  |  |  |  |  |  |  |
|  |  |  |  |  |  |  |  |  | 77.90%--slough |  |  |  |  |  |  |  |  |  |  |  |  |
| 9 | Zahia, S. | 2020 | CNN | 83.3% | 16.7% | NR | NR | NR | 87% | NR | NR | NR | NR | NR | NR | NR | NR | NR | NR | NR | NR |
