# Supplementary material for: Using Machine Learning Technologies in Pressure Injury Management: Systematic Review
Source: JMIR Med Inform. 2021 Mar 10;9(3):e25704. doi: 10.2196/25704 (PMC7991995; doi:10.2196/25704)
Supplement: Multimedia Appendix 2 [file medinform_v9i3e25704_app2.docx]

**Multimedia Appendix 2.** The detailed performance measurements of machine learning technologies in the included studies.

Table 2 summary of predictive model studies

| No. | author | year | country | aim | EHRs of patients | | | | | algorithm | risk factors | findings |
| --- | --- | --- | --- | --- | --- | --- | --- | --- | --- | --- | --- | --- |
|  |  |  |  |  | number | type | female (%) | age(year) | PI(%） |  |  |  |
| 1 | Su, Chao-Ton[59] | 2012 | China | To use data mining techniques to construct the prediction model for PIs. | 168 | surgery | 65.4% | PI:65.4±7.46 | 4.7% | MTS | age | Mahalanobis Taguchi System (MTS) can obtain robust and stable results. |
|  |  |  |  |  |  |  |  |  |  | SVMs | weight |  |
|  |  |  |  |  |  |  |  |  |  |  | surgery type |  |
|  |  |  |  |  |  |  |  |  |  | DT | anesthesia |  |
|  |  |  |  |  |  |  |  |  |  | LR | body position during the operation |  |
| 2 | Dai, L[40] | 2012 | China | To discuss the risk factors for PI of orthopedic patients. | 201 | orthopedics | 41.3% | 46.64±13.57 | 81.1% | NN | age | Logistic regression analysis combined with BP neural network shows, age, hemiplegia, external fixation and bone traction have a linear relationship with the logarithm advantage of the Braden classifications. |
|  |  |  |  |  |  |  |  |  |  |  | hemiplegic paralysis |  |
|  |  |  |  |  |  |  |  |  |  |  | external fixation |  |
|  |  |  |  |  |  |  |  |  |  |  | bone traction |  |
| 3 | Raju, D.[25] | 2015 | USA | To compare several data mining models to examine patient level factors associated with PIs. | 1653 | medical-surgical | 40.4% | 54±21.5 | 20% | LR | days in the hospital | Data mining, particularly, random forests are useful in predictive modeling. |
|  |  |  |  |  |  | critical care |  |  |  | DT | serum albumin |  |
|  |  |  |  |  |  | step-down units |  |  |  | RF | age |  |
|  |  |  |  |  |  |  |  |  |  | LR | blood urea nitrogen |  |
|  |  |  |  |  |  |  |  |  |  |  | Braden total score |  |
| 4 | Setoguchi, Y.[49] | 2016 | Japan | To develop a prediction model for PI cases that continue to occur at an acute care hospital with a low occurrence rate of Pus. | 8286 | hospitalization | 44.4% | PI  (female):67.2±14.7 | 0.62% | DT | transfer activity | The alternating DT method was effective in identifying factors within largely imbalanced data. |
|  |  |  |  |  |  |  |  |  |  |  | operation time |  |
|  |  |  |  |  |  |  |  |  |  |  | low BMI |  |
| 5 | Deng, X.H[41] | 2016 | China | To explore and evaluate the feasibility and accuracy of applying DT methods to predict the risk of hospital-acquired PIs in ICU patients. | 468 | ICU | 25.4% | 57.81±16.72 | 20.1% | DT | age | The DT model is an easy and feasible tool to predict the risk of hospital-acquired PIs in ICU patients, and it can be used to screen high-risk populations. |
|  |  |  |  |  |  |  |  |  |  |  | fecal incontinence |  |
|  |  |  |  |  |  |  |  |  |  |  | Braden total score |  |
|  |  |  |  |  |  |  |  |  |  |  | diastolic blood pressure |  |
| 6 | Kaewprag, P.[26] | 2017 | USA | To demonstrate that Bayesian network method is a powerful tool in inferring predictive models for syndromes such as PI from complex clinical data. | 7717 | ICU | 42.6% | 57.7±15.9 | 7.6% | BN | cardiovascular | The model gives comparable overall performance to the best of classical machine learning algorithms, while nearly tripling sensitivity at only a slight cost to specificity with no sacrifice on high overall accuracy. |
|  |  |  |  |  |  |  |  |  |  |  | kidney |  |
|  |  |  |  |  |  |  |  |  |  |  | lung |  |
|  |  |  |  |  |  |  |  |  |  |  | spinal cord |  |
|  |  |  |  |  |  |  |  |  |  |  | bladder |  |
| 7 | Chen, H. L.[39] | 2017 | China | To build an artificial NN model for predicting surgery-related PI in cardiovascular surgical patients. | 147 | cardiovascular surgery and aortic surgery | 46.9% | 49.8±17.7 | 24.8% | NN | age | The artificial NN model that provides moderate prediction of surgery-related PI in patients undergoing cardiovascular surgical procedures. |
|  |  |  |  |  |  |  |  |  |  |  | disease category |  |
|  |  |  |  |  |  |  |  |  |  |  | surgery duration |  |
|  |  |  |  |  |  |  |  |  |  |  | perioperative corticosteroids administration |  |
| 8 | Moon, M[53] | 2017 | Korea | To use DT analysis to explore the factors associated with PIs among elderly people admitted to Korean long-term care facilities. | 15856 | long-term care facilities | 70.9% | 81 | 50% | DT | length of stay | The data mining methods could identify outcome variables in a big data set with many variables. |
|  |  |  |  |  |  |  |  |  |  |  | comorbidity |  |
|  |  |  |  |  |  |  |  |  |  |  | total hospital cost |  |
| 9 | Alderden, J.[27] | 2018 | USA | To develop a model for predicting development of PIs among surgical critical care patients. | 6376 | surgical ICU | 37.7% | 54±19 | 8.1% | RF | BMI | The AUC of the developed model to predict risk for PIs among critical care patients is 0.79. |
|  |  |  |  |  |  |  |  |  |  |  | hemoglobin level |  |
|  |  |  |  |  |  |  |  |  |  |  | creatinine level |  |
|  |  |  |  |  |  |  |  |  |  |  | time required for surgery |  |
|  |  |  |  |  |  |  |  |  |  |  | age |  |
| 10 | Yang, Q[42] | 2019 | China | To evaluate the accuracy and rationality of DT in predicting the risk of unavoidable PIs for cancer patients. | 611 | cancer | 35.5% | 61.8±13.6 | 7.5% | DT | Braden total score | The DT model enjoys good area under the ROC curve, sensitivity and specificity, making it suitable to screen and manage cancer patients at high risk of unavoidable PIs. |
|  |  |  |  |  |  |  |  |  |  |  | existing or potential skin damage |  |
|  |  |  |  |  |  |  |  |  |  |  | no repositioning plan was implemented |  |
|  |  |  |  |  |  |  |  |  |  |  | special situations increasing risk of PU existed |  |
| 11 | Li, H. L.[37] | 2019 | China | To investigated the association between patient characteristics and the occurrence of PIs for patients at the end of life. | 2062 | at the end of life | 45.4% | 75.5±14.4 | 49.8% | LR | history of PIs | The LR and the DT algorithm provided the most interpretable models. |
|  |  |  |  |  |  |  |  |  |  | SVMs | without cancer |  |
|  |  |  |  |  |  |  |  |  |  | DT | excretion |  |
|  |  |  |  |  |  |  |  |  |  | NN | activity/mobility |  |
|  |  |  |  |  |  |  |  |  |  |  | skin condition/circulation |  |
| 12 | Sprigle, S[33] | 2020 | USA | To assess PI risk in persons with mobility impairments using a large data set to identify demographic, laboratory, hemodynamic, and pharmacologic risk factors. | 1252313 | mobility-related disabilities | 54.1% | unclear | 6.9% | gradient boosting | Alzhemier’s disease | Although persons with disabilities can exhibit a wide functional range, they remain at risk of PIs and should be evaluated for proper preventive measures, including support surfaces and wheelchair cushions. |
|  |  |  |  |  |  |  |  |  |  |  | cerebral palsy |  |
|  |  |  |  |  |  |  |  |  |  |  | hemiplegia |  |
|  |  |  |  |  |  |  |  |  |  |  | multiple sclerosis |  |
|  |  |  |  |  |  |  |  |  |  |  | paraplegia/quadriplegia |  |

Table 3 summary of posture recognition studies

| No. | author | year | country | aim | sensor | | subject | | algorithm | posture/movement | findings |
| --- | --- | --- | --- | --- | --- | --- | --- | --- | --- | --- | --- |
|  |  |  |  |  | number | detail | number | detail |  |  |  |
| 1 | Barsocchi, P.[51] | 2012 | Italy | To propose a system able to automatically assess the PI risk. | 4 | 3 fixed sensors placed in a typical bedroom | 2 | female (1.68m,63kg) | k-NN | right lateral | A simple k-NN classifier performs better than a more sophisticated SVM classifier. |
|  |  |  |  |  |  |  |  |  |  | left lateral |  |
|  |  |  |  |  |  |  |  |  |  | supine |  |
|  |  |  |  |  |  | a mobile sensor placed on the subject breast |  | male (1.78m,95kg) | SVM | prone |  |
|  |  |  |  |  |  |  |  |  |  | 30° lateral |  |
| 2 | Baran Pouyan, M.[29] | 2014 | USA | To develop a novel and robust methodology for estimating bed inclination by use of a low-resolution pressure mat. | 2048 | a commercial pressure mat | 15 | different sizes and weights | k-NN | 0 degree | The method predicts bed inclination in three classes with 80.3% average accuracy. |
|  |  |  |  |  |  |  |  |  | Naïve-bayes | 30 degree |  |
|  |  |  |  |  |  |  |  |  | DT | 60 degree |  |
| 3 | Xu, X.[34] | 2016 | China | To propose a matching-based approach---BEMD, for sleep posture recognition. | 8192 | smart bedsheet system | 14 | male (9) | k-NN | left-log | The achieved accuracy of 91.21% outperforms the previous work with an improvement of 8.01%. |
|  |  |  |  |  |  | pressure sensor array |  | female (5) |  | left-fetus |  |
|  |  |  |  |  |  | a data sampling unit |  | weight (55-85kg) |  | right-log |  |
|  |  |  |  |  |  |  |  |  |  | right-fetus |  |
|  |  |  |  |  |  | a terminal for data analysis and storage |  | height (155-185cm) |  | supine |  |
|  |  |  |  |  |  |  |  |  |  | prone |  |
| 4 | Baran Pouyan, M.[23] | 2016 | USA | To propose a graph-based clustering approach to extract the body limbs from the pressure data collected by a commercial pressure map system. | 1728 | a commercial non-invasive pressure-sensitive mat platform | 15 | different weights, heights and ages | k-NN | right lateral | The proposed approach has high performance and more than 94% average accuracy. |
|  |  |  |  |  |  |  |  |  |  | left lateral |  |
|  |  |  |  |  |  |  |  |  |  | supine |  |
| 5 | Heydarzadeh, M.[30] | 2016 | USA | To apply deep autoencoder neural networks to the histogram of gradient (HoG) of the pressure image. | 2048 | a flexible mat with resistive sensors uniformly distributed | 10 | NR | NN | right foetus | High accuracy of up to 98% was achieved in classifying five different in-bed postures for more than 60,000 pressure images. |
|  |  |  |  |  |  |  |  |  |  | right yearner |  |
|  |  |  |  |  |  |  |  |  |  | supine |  |
|  |  |  |  |  |  |  |  |  |  | left yearner |  |
|  |  |  |  |  |  |  |  |  |  | left foetus |  |
| 6 | Hsiao, R. S.[35] | 2016 | China | To propose body posture recognition and turning recording system for assisting the care of bed bound patients in nursing homes. | 15 | body posture recognition and turning recording system | 9 | 3 different body-weight | SVM | right lateral | The average posture recognition accuracy for the proposed module is 92%. |
|  |  |  |  |  |  |  |  | small (40kg) | Fuzzy | left lateral |  |
|  |  |  |  |  |  |  |  | medium (60kg) | k-NN | supine |  |
|  |  |  |  |  |  |  |  | larger(80kg) |  |  |  |
| 7 | Ma, C.[36] | 2017 | China | To design a wheelchair assist system for mobility-impaired individuals that can recognize postures using a cushion-based system. | 12 | smart cushion for wheelchair users | 12 | female (5) | DT | proper sitting | The proposed sensor deployment is effective, achieving 99.47% posture recognition accuracy. |
|  |  |  |  |  |  |  |  | 3 different BMI distribution | SVM | lean left |  |
|  |  |  |  |  |  |  |  | underweight（4） | NN | lean right |  |
|  |  |  |  |  |  |  |  | normal (4) | Naïve Bayes | lean forward |  |
|  |  |  |  |  |  |  |  | overweight and obese (4) | k-NN | lean backward |  |
| 8 | Enayati, M.[32] | 2018 | USA | To evaluate different parameter settings for the application of neural networks in sleep posture detection using the data acquired from an in-home setting of only four hydraulic bed sensors. | 4 | hydraulic bed transducers placed underneath the mattress | 58 | young healthy subjects | NN | supine | There are multiple configuration settings that make classification accuracy as high as 100% using k-Fold CV for all postures. |
|  |  |  |  |  |  |  |  |  |  | prone |  |
|  |  |  |  |  |  |  |  |  |  | left lateral |  |
|  |  |  |  |  |  |  |  |  |  | right lateral |  |
| 9 | Matar, G[31]. | 2018 | Canada | To propose an autonomous method for classifying the four state-of-art HBLPs in healthy adults. | 1728 | a pressure sensor mattress | 12 | Female (2) | NN | supine | The results are satisfactorily promising towards unobtrusively monitoring posture for ulcer prevention. |
|  |  |  |  |  |  |  |  |  |  | prone |  |
|  |  |  |  |  |  |  |  | age:27.35±5.39 |  | right lateral |  |
|  |  |  |  |  |  |  |  |  |  | left lateral |  |
| 10 | Duvall, J.[24] | 2019 | USA | To evaluate the feasibility of using load cell technology (E-Scale) to support PI prevention by determining whether load cells can provide insight into when and what type of position changes occur for an individual in bed. | 4 | E-scale placed under the bed legs | 10 | able-bodied | k-NN | rolls | The E-scale was able to detect and classify four types of movements with>94% accuracy. |
|  |  |  |  |  |  |  |  |  |  | turns in place |  |
|  |  |  |  |  |  |  |  |  |  | extremity movements |  |
|  |  |  |  |  |  |  |  |  |  | assisted turns |  |
|  |  |  |  |  |  |  |  |  |  | all |  |
| 11 | Cicceri, G[52] | 2020 | Italy | To monitor the patient with wearable sensors with the support of machine learning techniques in order to estimate his/her position over time and assess the risk of PIs formation. | NR | a set of wearable sensors including three-axis accelerometer, a three-axis magnetometer | 6 | different age, weight, and sex | DNN | supine | Deep learning techniques give better performance compared to other machine learning techniques like SVMs and RF, and can be considered reliable support for patient health also in the domain of PIs prevention. |
|  |  |  |  |  |  |  |  |  |  | prone |  |
|  |  |  |  |  |  |  |  |  | SVM | right |  |
|  |  |  |  |  |  |  |  |  |  | left |  |
|  |  |  |  |  |  |  |  |  | RF | sitting |  |
|  |  |  |  |  |  |  |  |  |  | movement |  |

Table 4 summary of image analysis studies

| No. | author | year | country | aim | image | | | algorithm | outcome | findings |
| --- | --- | --- | --- | --- | --- | --- | --- | --- | --- | --- |
|  |  |  |  |  | number | from | detail |  |  |  |
| 1 | Kosmopoulos, D.[54] | 2007 | Greece | To examine the potential of using digital images to classify regions appearing in PI images. | 85 | various hospitals | each of them provided as many as 50 segments and provided samples of different classes | SVM | stage 1 | Most misclassifications occurred between close stages. |
|  |  |  |  |  |  |  |  |  | stage 2 |  |
|  |  |  |  |  |  |  |  |  | stage 3 |  |
|  |  |  |  |  |  |  |  |  | stage 4 |  |
|  |  |  |  |  |  |  |  |  | white necrosis |  |
|  |  |  |  |  |  |  |  |  | black necrosis |  |
| 2 | Veredas, F. J.[47] | 2010 | Spain | To design a computational approach to tissue recognition in PI images. | 113 | sacrum and hip PIs of 69 patients with home -care assistance | Canon EOS 40D digital camera | NN | granulation | The binary cascade approach gives high global performance rates and shows the highest average sensitivity score when detecting necrotic tissue in the wound. |
|  |  |  |  |  |  |  | Sigma EM-140 ring light |  | slough |  |
|  |  |  |  |  |  |  | a distance of approximately 30-40cm from the wound plane |  | necrosis |  |
|  |  |  |  |  |  |  |  | Naïve-Bayes | healing |  |
|  |  |  |  |  |  |  |  |  | skin |  |
| 3 | Noguchi, H.[50] | 2014 | Japan | To confirm the feasibility of clustering technique for discovery of specific local pattern in the stained image by comparison of human classification and to evaluate the performance of machine classification on nurse's supervised images. | 14 | LumiCube | average size was about 1000 pixel square  different wound sizes | k-means | dot | As for the clustering, three features achieved the similar performance. As for the classification based on human-annotated images, LBP and wavelets showed good performance. |
|  |  |  |  |  |  |  |  |  | cloud |  |
|  |  |  |  |  |  |  |  | SVMs | plane |  |
|  |  |  |  |  |  |  |  |  | background |  |
| 4 | Veredas, F. J.[44] | 2015 | Spain | To present a computer-vision approach to wound-area detection based on statistical color models. | 113 | sacrum and hip PIs of 69 patients with home -care assistance | 1224*1632 | k-means | granulation | The final fitted segmentation model shows robustness and gives high mean performance rates when segmenting significant wound areas that include healing tissues. |
|  |  |  |  |  |  |  | Sony Cybershot W30 digital camera |  | slough |  |
|  |  |  |  |  |  |  | a distance of approximately 30-40cm from the wound plane |  | necrosis |  |
|  |  |  |  |  |  |  | under non-controlled illumination conditions |  | periphery |  |
|  |  |  |  |  |  |  | JEPG format |  |  |  |
| 5 | Veredas, F. J.[46] | 2015 | Spain | To present a computer-vision approach based on image processing algorithms and supervised learning techniques to help detect and classify wound tissue types that play an important role in wound diagnosis. | 113 | sacrum and hip PIs of 69 patients with home -care assistance | Sony Cybershot W30 digital camera | NN | granulation | SVMs and RF gave the high performance rates when classifying wound-bed patterns composed of color, texture, region morphology and topology features extracted from the segmented regions in a set of real PI images. |
|  |  |  |  |  |  |  | a distance of approximately 30-40cm from the wound plane | SVMs | slough |  |
|  |  |  |  |  |  |  |  | RF | necrotic |  |
|  |  |  |  |  |  |  | under non-controlled illumination conditions |  | healing |  |
| 6 | Li, D.[28] | 2017 | USA | To develop an image processing algorithm to automatically measure PIs using electronic PI images stored in nursing documentation. | 32 | the local hospital nursing documentation | different camera manufactures, settings and light condition | SVMs | length | The image processing algorithm is a reliable approach to monitoring PI progress through clinical PI images. |
|  |  |  |  |  |  |  |  |  | width |  |
|  |  |  |  |  |  |  |  |  | surface area |  |
| 7 | Garcia-Zapirain, B.[43] | 2018 | Spain | To propose an automated segmentation system to segment and classify different tissues from PI colored images. | 193 | IGURCO GESTION S.L. (36) | 1024*1024 | CNN | granulation | The obtained preliminary DSC of 92%, PAD of 13%, and AUC of 95% are promising. |
|  |  |  |  |  |  | Medetec wound database (157) | 1024*731 |  | necrotic eschar |  |
|  |  |  |  |  |  |  |  |  | slough |  |
| 8 | Zahia, S.[45] | 2018 | Spain | To perform optimized segmentation of the different tissue types present in PIs. | 22 | the Igurko hospital (18) | 1020*1020 | CNN | granulation | The approach shows an overall average classification accuracy of 92.01%, an average total weighted DSC of 91.38%, and an average precision per class of 97.31% for granulation tissue, 96.59% for necrotic tissue, and 77.90% for slough tissue. |
|  |  |  |  |  |  |  |  |  | necrosis |  |
|  |  |  |  |  |  | the NPUAP online store (4) | using flash |  | slough |  |
| 9 | Zahia, S.[48] | 2020 | Spain | To propose an end-to-end system which automatically segments the PI and extracts all quantitative information by matching the 2D image with its 3D mesh. | 210 | several Spanish hospitals  (110) | 3D using the Struture Sensor | CNN | depth | The proposed framework can not only output refined segmentation with 87% precision, but also retrieves reliable measurements, which can be used for medical assessment and healing evaluation of PIs. |
|  |  |  |  |  |  |  | 2D using a cell-phone camera |  | area |  |
|  |  |  |  |  |  | Medetec Mediacl Images online database  (100) | resolution form 1080*2280 to 2747*3079 |  | volume |  |
|  |  |  |  |  |  |  | No flash light used |  | major and minor axes |  |
